# Nanoscale uniformity in the active tuning of a plasmonic array by polymer gel volume change†

**DOI:** 10.1039/c8na00404h

**Published:** 2019-03-04

**Authors:** Satoru Hamajima, Hideyuki Mitomo, Takeharu Tani, Yasutaka Matsuo, Kenichi Niikura, Masayuki Naya, Kuniharu Ijiro

**Affiliations:** Graduate School of Chemical Sciences and Engineering, Hokkaido University Kita 13, Nishi 8, Kita-Ku Sapporo 060-8628 Japan; Research Institute for Electronic Science, Hokkaido University Kita 21, Nishi 10, Kita-Ku Sapporo 001-0021 Japan mitomo@poly.es.hokudai.ac.jp; Global Station for Soft Matter, Global Institution for Collaborative Research and Education, Hokkaido University Kita 21, Nishi 11, Kita-Ku Sapporo 001-0021 Japan; FUJIFILM Corporation Ushijima, Kaisei-Machi, Ashigarakami-gun Kanagawa 258-8577 Japan; Department of Applied Chemistry, Faculty of Fundamental Engineering, Nippon Institute of Technology Miyashiro Saitama 345-8501 Japan

## Abstract

Active plasmonic tuning is an attractive but challenging research subject, leading to various promising applications. As one of the approaches, nanostructures are placed in or on soft matter, such as elastomers and gels, and their gap distances are tuned by the mechanical extension or volume change of the supporting matrices. As hydrogels possess various types of stimuli-responsiveness with large volume change and biocompatibility, they are good candidates as supporting materials for active nanostructure tuning. However, it remains unclear how accurately we can control their nanogap distance changes using polymer gels with a low deviation due to major difficulties in the precise observation of nanostructures on the gels. Here, we prepared gold arrays with sub-100 nm dots on silicon substrates by electron beam lithography and transferred them onto the hydrogel surface. Then, their nanopattern was actively tuned by the changes in gel size in water and their structural changes were confirmed by optical microscopy, microspectroscopy, and atomic force microscopy (AFM). Further, we successfully prepared ionic liquid (IL) gels with various degrees of swelling *via* solvent exchange. Scanning electron microscopy (SEM) observation of the IL gels provided clear pictures at nanoscale resolution. Finally, we calculated the plasmonic spectra using a finite difference time domain (FDTD) simulation based on the SEM images and compared them with the measured spectra. The results in this study totally support the notion that active changes in plasmonic nanodot patterns *via* volume changes in the hydrogel are quite homogenous on a several nanometer scale, making them ideal for precise active surface plasmon tuning.

## Abstract 2

Nanoscale imaging and spectral analyses support the tremendous potential of the active tuning of nanostructures by hydrogels.

## Graphical abstract

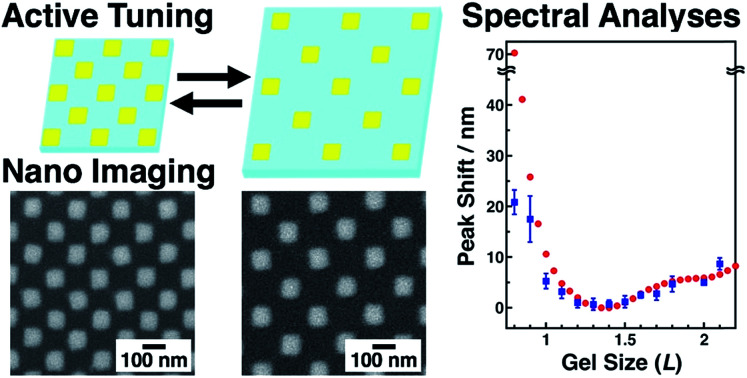

## Introduction

Metal nanostructures have attracted a great deal of attention due to their potential applications in optical or photonic devices based on surface plasmon resonance (SPR), which is induced by strong light–metal interactions.^1–4^ Recently, the active control of SPR is a new and challenging field known as active plasmonics.^5–8^ Development of an actively tunable SPR enables the on-demand enhancement of optical signals and further advances in the application of plasmonic materials, such as subwavelength photonic devices and plasmonic sensing.^9–12^ Major categories of active plasmon control include plasmonic structures in tunable surroundings and those with tunable gap distances, as SPR is sensitive to geometrical parameters, spatial arrangements, and surrounding dielectric environments.^5,8,13^ Changes in the environment surrounding plasmonic structures have been studied well as they are beneficial for the quantitative analysis of various chemicals and biomolecules.^14–16^ On the other hand, although it remains challenging, active tuning of the plasmonic nanostructures is thought to have greater potential due to the plasmon coupling effects, which are induced when the metal nanostructures are in close proximity, as a new plasmon resonance mode with large electromagnetic field enhancements.^7,13,17,18^

Plasmonic structures with tunable gap distances have been constructed by the integration of metal nanoparticles with some molecules or polymers that provide specific interactions for reversible assembly or possess stimuli-responsive conformational changes.^8,19^ They are roughly classified into two approaches. In the first, surface-functionalized nanoparticles are dispersed in solution. There are many reports that metal nanoparticles modified with stimuli-responsive molecules reversibly assemble in solutions in response to external stimuli such as pH,^20–24^ temperature,^25–27^ or light,^28,29^ providing dynamic tuning of plasmon coupling. However, it is still difficult to precisely control assembled structures including their assembly size and gap distances. To overcome these issues, new approaches, such as an external molecule- or polymer-assisted co-assembly, have been under development.^30,31^ In the other approach, nanostructures are placed in or on soft matter, such as elastomers and gels, and their gap distances are tuned by the mechanical extension or volume change of the supporting matrices. So far, these polymer-supported approaches have shown better results compared to the assembly–disassembly control as a dispersion in solutions from the viewpoint of reproducibility and adjustability. The most popular among this type of approach, involves poly(dimethylsiloxane) (PDMS), an elastomer that is often used as a supporting substrate due to its transparency, extensibility, and ease of handling. PDMS-based active plasmonic substrates have been widely applied to fundamental research on SPR^32–35^ and surface-enhanced Raman scattering (SERS)^36,37^ and applications such as tunable coloring materials.^38–40^ Although SERS is one of the promising applications for label-free detection of biomolecules such as proteins with high sensitivity by the extreme enhancing effect of Raman scattering signals, which is known as intrinsic chemical fingerprint information, at the enhanced electromagnetic field on SPR, there are few reports on this, probably due to the unsuitable affinity between PDMS and biomolecules. From the viewpoint of compatibility with biomolecules, hydrogels are thought to be a better substrate. However, the optimization of nanostructures on the hydrogels is very limited due to technical problems related to the preparation and observation of ordered metal nanoarrays on the hydrogels, although the precise control of ordered nanostructures is important to achieve better performance on active plasmonics.^41–45^

In our previous study, we developed a method for preparing metal nanostructures on hydrogels.^46^ In this method, fine gold structures prepared by lithographic techniques on a silicon substrate were transferred onto a poly(acrylic acid) (PAA) gel based on the moderate interactions between the gold and gel. The intervals of the fine structures on the hydrogels were actively tuned *via* their volume changes. Optical microscopy imaging supported their structural information on a micrometer scale. Further, metal nanostructures, which were prepared *via* self-assembly of metal nanoparticles, were also transferred onto the PAA gel. This worked as an active plasmonic substrate and provided a new insight into SERS substrates with active gap control.^47^ The active gap control system could intensify the SERS signals from proteins as a macromolecule due to the coexistence of efficient incorporation of analytes into the widened gaps as an open form and the greater enhancing effect on SPR with narrow gaps as a closed form. Although these reports indicate significant potential for hydrogel-based SERS substrates, a number of issues remain to be clarified including the uniformity of the structural change on a nanoscale, as there are some reports on the inhomogeneity of the polymer networks in the gels on the scale of tens to hundred nanometers.^48,49^

**Fig. 1:**
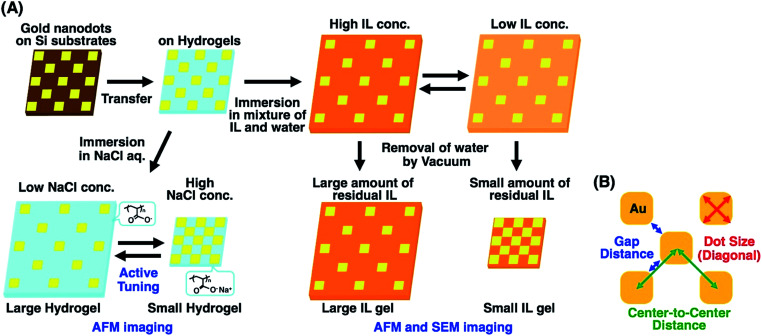
Preparation scheme of tuned gold nanodot arrays on hydrogels and ionic liquid gels with various swelling degrees (A) and the sizes determined for the evaluation of structural changes (B).

To address this issue, in this study, we prepared gold nanoarrays composed of sub-100 nm dots on the hydrogels and performed nanoscale imaging with various swelling degrees by atomic force microscopy (AFM) and scanning electron microscopy (SEM) (Fig. 1A). As mentioned elsewhere, direct imaging of nanostructures on a hydrogel has some difficulties or limitations. AFM provided direct images of the nanopattern on the hydrogels in water at a lower resolution. As SEM doesn't work for the direct imaging of swollen hydrogels due to their shrinkage under vacuum, SEM imaging was performed after solvent exchange from water to a nonvolatile ionic liquid (IL). SEM imaging of IL gels provided clear pictures at the nanoscale resolution. Based on the SEM images, we evaluated the structural change in nanodot arrays by determining the center-to-center distances, gap distances, and dot sizes (Fig. 1B). Also finite difference time domain (FDTD) simulation was performed based on the SEM images and compared with plasmonic spectra from experimental measurements. These results totally supported the nanoscale uniformity in active tuning of the plasmonic nanoarrays using volume changes in the polymer gels, even when simply prepared by free radical polymerization. This uniform change in nanoscale enables the precise control of plasmonic nanostructures, leading to improved active plasmonic functions.

## Experimental

### Materials

16-Amino-1-hexadecanethiol hydrochloride was purchased from Dojindo Laboratories (Japan). Acrylic acid, tetra(ethylene glycol)diacrylate, and 2-oxoglutaric acid were purchased from Tokyo Chemical Industry Co., Ltd. (Japan). Acrylic acid was used after purification by vacuum distillation. Hexa(ethylene glycol)undecanethiol was synthesized according to our previous report.^50^ NaCl was purchased from FUJIFILM Wako Pure Chemical Corp. (Japan). 1-Methylimidazolium hydrogen sulfate was purchased from Sigma-Aldrich Co. LLC. (USA).

### Preparation of gold nanodot arrays on the hydrogels

Silicon wafers were initially cleaned with a piranha solution. An electron beam (EB) resist ZEP520A (Zeon Corp., Japan) was coated on the silicon wafers using a spin coater (MS-A150, Mikasa Co., Ltd., Japan). EB exposure was performed using an EB writing system (ELS-F125-U, Elionix Inc., Japan). After exposure, wafers were immersed in developer solution ZMD-N50 (Zeon Corp., Japan). A gold film of 30 nm thickness was deposited using a sputtering apparatus (MPS-4000C1/HC1, ULVAC Inc., Japan), followed by a lift-off process in remover solution ZDMAC (Zeon Corp., Japan). The gold nanodot pattern prepared on a silicon wafer was observed using a reflection microscope (VK-9710, KEYENCE Corp., Japan) and SEM (JSM-6700FT, JEOL Ltd., Japan). SEM images were obtained with 5 kV accelerating voltage. Gold nanodot surfaces were exposed to 1 mM of 16-amino-1-hexadecanethiol in 1-propanol. An aqueous solution containing acrylic acid (2 M), tetra(ethylene glycol)diacrylate (10 mM), and 2-oxoglutaric acid (8 mM) was prepared as a monomer solution for PAA gels. After removal of dissolved oxygen by nitrogen bubbling for 10 minutes, the monomer solution was poured into the mold, which is prepared with a glass slide, 1 mm thick silicone spacer, and the silicon wafer with gold nanodots. Then, PAA gel was synthesized by a free radical polymerization under UV irradiation with an UV lamp (XX-15BLB, Ultra-Violet Products Ltd., Britain) for 6 hours. The prepared gels were gently released from the molds. The transfer of gold nanodot arrays from silicon wafers to the gel surface was confirmed by microscopic observation. The PAA gel with gold nanodots was immersed in an aqueous solution containing hexa(ethylene glycol)undecanethiol (1 mM) and NaCl (500 mM) for 60 minutes for the modification of newly exposed gold nanodot surfaces, which had been in contact with the silicon wafer.

### Active tuning of gold nanodot arrays by volume change of hydrogels

After removal of unbounded surface ligands, PAA gels were sequentially immersed in NaCl aqueous solution with various concentrations to change their volumes. The gold nanodot arrays on the gels with various swelling degrees were observed using a transmission microscope (BX51, Olympus Corp., Japan) and AFM (MFP-3D-BIO-J, Oxford Instruments plc, Britain) in solution. AFM imaging was performed in the AC mode with a silicon nitride cantilever (BL-AC40TS, Olympus Corp., Japan). Extinction spectra of the gold nanodot arrays on PAA hydrogel surfaces were measured using an UV-Vis-NIR microspectrometer (MSV-5200, JASCO Corp., Japan).

### SEM observation using an ionic liquid

The PAA hydrogels with gold nanodot arrays were immersed in a mixture of 1-methylimidazolium hydrogen sulfate and ultrapure water for 3 hours, and further immersed in a fresh mixture for 3 hours. This immersion process replaced the solvent of PAA gels from NaCl aqueous solution to the mixture of IL and water. After immersion, the gel was placed on transparent conductive glass (GEOMATEC Co., Ltd., Japan). Water contained in the PAA gel swollen with the mixture was evaporated using a vacuum using oil-sealed rotary pump (GLD-136C, ULVAC Inc., Japan) for 15 hours. The gold nanodot arrays on the ionic liquid gel surface were observed using an optical microscope and AFM in air. Extinction spectra of the gold nanodot array on the ionic liquid gel surface were measured using an UV-Vis-NIR microspectrometer in air. Then, SEM observations were performed with 5 kV accelerating voltage.

### Calculation of extinction cross-section spectra by FDTD simulation

The optical properties of gold nanodot arrays were calculated by employing an electromagnetic optical simulation based on the finite difference time domain (FDTD) method. The shape of the dot was approximated by a rounded square prism. From the top view of the SEM image, the length of one side was 80.4 nm and the radius of the corner was set to 21 nm. Thickness was 30 nm. We modeled the basic unit structure and calculated under periodic boundary conditions. The length of the period was changed according to the change of pitch. The surrounding medium was pure water. The refractive index of gold was based on previously published data.^51^ The incident light was a plane wave of normal incidence and circular polarization. Then, the straight transmittance was calculated.

## Results and discussion

### Preparation of gold nanodot arrays on the hydrogels

**Fig. 2:**
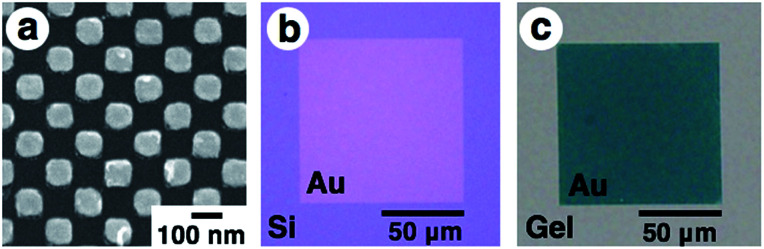
Scanning electron microscopic image of the prepared gold array with sub-100 nm square dots on a silicon substrate (a), optical microscopic images of the gold nanodot array with 100 × 100 μm in area on silicon before transfer (b) and on a gel after transfer (c). Due to the different transparencies of the substrates, optical microscopic imaging was performed in different modes. (b) is a reflection image and (c) is a transmission image. Au represents the area of the gold nanodot array, which looks gold in reflection mode (b) and green in transmittance mode (c).

First, gold nanodot arrays were prepared on silicon substrates by the conventional electron beam (EB) lithographic method and transferred onto the PAA gel surface (Fig. 2).^46^ In this study, we designed an array with 80 × 80 nm square dots. Details of the original design (CAD image) are shown in Fig. S1.†Fig. 2a shows a scanning electron microscopic (SEM) image of the prepared nanodot pattern on a silicon substrate. The dot size as a diagonal length, the center-to-center distance between nanodots, and the gap distance between the nearest edges were determined from these images using the ImageJ 1.51j program as 101 ± 5, 138 ± 2, and 36 ± 5 nm, respectively (summarized in Table 1). Although a little blunting of their shape was observed due to technical issues with the EB lithography, a low value for the standard deviation of the center-to-center distance (less than 2% of relative standard deviation) supported the fact that a highly uniform gold pattern was prepared. Then, 16-amino-1-hexadecanethiol solution was dropped onto the gold pattern for surface modification to provide electrostatic interaction with the acidic gels. After self-assembled monolayer formation, PAA gels were prepared on this substrate by conventional free radical polymerization. Finally, gold arrays were efficiently transferred onto the PAA hydrogel surface with the aid of strong electrostatic interactions (Fig. 2b and c).

**Table 1:** Center-to-center distances, gap distances, and dot sizes as the diagonal length of the nanodot pattern on the IL gels for various gel size (*L*) determined from SEM images^a^

Gel size (*L*)	0.8	1.0	1.1	1.3	1.5	On silicon as a reference
Center-to-center distance (nm)	108 ± 7 (*n* = 100)	138 ± 6 (*n* = 64)	144 ± 4 (*n* = 64)	176 ± 5 (*n* = 36)	211 ± 10 (*n* = 36)	138 ± 2 (*n* = 64)
Gap distance (nm)	15 ± 6 (*n* = 144)	42 ± 6 (*n* = 100)	50 ± 6 (*n* = 100)	78 ± 6 (*n* = 64)	116 ± 11 (*n* = 64)	36 ± 5 (*n* = 100)
Dot size as the diagonal length (nm)	95 ± 4 (*n* = 122)	96 ± 4 (*n* = 82)	95 ± 5 (*n* = 82)	100 ± 3 (*n* = 50)	96 ± 5 (*n* = 50)	101 ± 5 (*n* = 82)

a Numbers correspond to the average ± standard deviation with the number of measurements shown in parentheses.

### Active tuning of gold nanodot arrays by hydrogel volume change

**Fig. 3:**
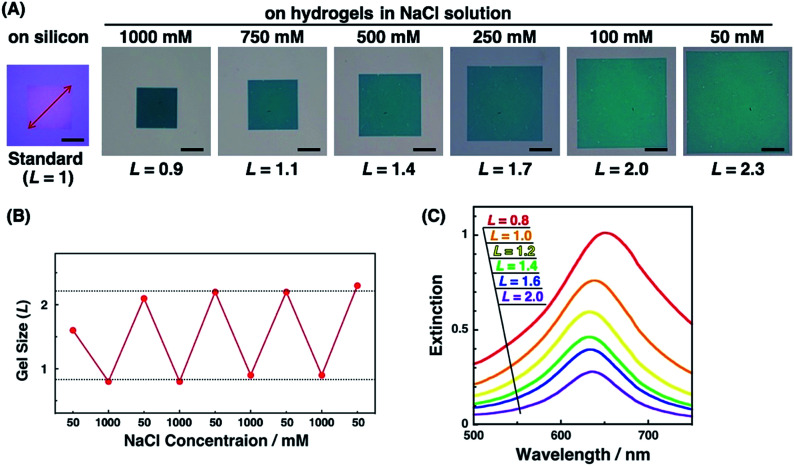
(A) Optical microscopic images of nanodot arrays on silicon and hydrogels with various swelling degrees, (B) reversible gel size (*L*) changes with the NaCl concentration, and (C) extinction spectra of nanodot arrays on hydrogels with various swelling degrees. Scale bars represent 50 μm (A).

Active tuning of plasmonic nanopatterns on the hydrogel was investigated by spectral measurement and AFM. When PAA gels were immersed in NaCl aq. solutions of various concentrations (50, 100, 250, 500, 750, and 1000 mM NaCl), the size of the gels changed depending on the salt concentration. The total patterned area also changed with the gel size (Fig. 3A). This change was reversible once the gel was completely shrunk in 1000 mM NaCl solution (Fig. 3B). As-prepared polymer gels often show these kinds of hysteresis, particularly on mechanical stretching. Thus, one possible reason in this case is that the polymer networks in the gel were not fully equilibrated at the first point in the 50 mM NaCl solution, even though it was immersed for several hours in the solution. Once the gel was immersed in the 1000 mM NaCl solution, it reached a fully shrunken state and reached an equilibrated network structure. For quantitative analyses, a relative gel size, referred to as *L*, was defined by the following equation based on the diagonal length of the total patterned area.*L* = (diagonal length of the total patterned area on the PAA gel)/(diagonal length of the total patterned area on a silicon substrate which represented the original size)

Next, we measured the extinction spectra of the gold nanopattern on the PAA gel at various sizes. Fig. 3C shows that the extinction spectra gradually changed depending on the gel size *L*. Extinction was decreased with the increase in the *L* value. This phenomenon is the same as our previous results and appears to be quite reasonable as the number of Au dots in the measured area decreased with increase in the gel size.^47^ An important result was observed in the spectral shifts. The peak wavelengths of each sample were plotted against *L* values (Fig. S2†). They showed a 10–20 nm difference in peak wavelength, even for the same *L* value, probably due to the small differences in the nanodot shape among samples prepared by EB lithography. To eliminate this discrepancy, we plotted Δ*λ*_*L*_ as the peak shift at gel size *L* from *λ*_min_ as the shortest wavelength of the sample (Fig. 8B blue; these data are compared to FDTD simulation data below). These plots show similar peak shifts depending on the gel size *L* with smaller deviations compared to those in Fig. S2.† Blue shifts in the plasmonic peak from *L* = 2.1 to 1.4 and red shifts from *L* = 1.3 to 0.8 were observed. These shifts are thought to result from plasmon coupling effects due to the close proximity of the gold nanodots. This is supported by the FDTD simulation results shown below and details of these spectral shifts are discussed below. In any case, the plasmonic peaks were actively tuned by the change in the salt concentration as shown in our previous report using gold nanoparticle assembled films.^47^

**Fig. 4:**
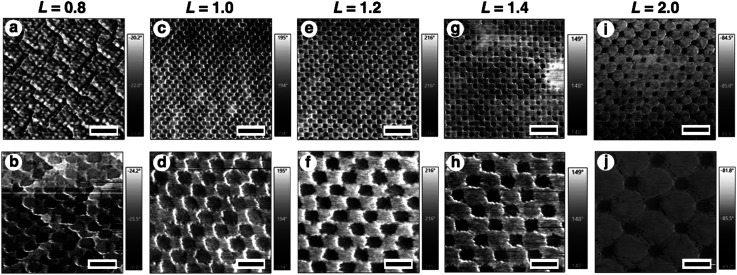
AFM phase images of the surface patterns on hydrogels of various sizes, *L* = 0.8 (a and b), *L* = 1.0 (c and d), *L* = 1.2 (e and f), *L* = 1.4 (g and h), and *L* = 2.0 (i and j). Scale bars represent 600 nm in the upper images (a, c, e, g and i) and 200 nm in the lower images (b, d, f, h and j).

There is no doubt that the gold nanodot patterns were actively changed by the volume change in the hydrogels. However, their actual structures in this active tuning remain unclear. Thus, we tried direct imaging of their structures on the hydrogel. There are two general approaches for observing nanoscale structures. One is scanning probe microscopy (SPM) and the other one is electron microscopy. As the size and/or shape of the hydrogels change under vacuum even when using lyophilization, it is normally unacceptable to apply electron microscopic imaging. AFM, which is an element of SPM, is a useful approach for observing the samples in water under ordinary pressure. Therefore, we obtained nanoscale images of the samples using AFM (Fig. 4 and S3†). The differences in characteristics between gold and hydrogels allow us to obtain clear phase images with good contrast. AFM images show that the gold nanodot patterns, represented as black areas, changed uniformly depending on the gel size *L*. In the case of a tightly shrunk gel (*L* = 0.8), the gold nanodots looked to be almost in contact and there were some wrinkles on the surface in the large-scale image (Fig. 4a and S3a†). These kinds of wrinkle formations are well-known.^52,53^ This wrinkle formation for *L* = 0.8 indicates that there was significant lateral pressure or stress on the gel surface during gel compaction. Here, we observed changes in the total gold nanodot patterned areas by optical microscopy, plasmonic spectra by microspectroscopy, and intervals by AFM depending on the volume changes in the hydrogels. However, we could not obtain AFM images with sufficient resolution for a plasmonic spectrum simulation due to technical difficulties.

### SEM observation using an ionic liquid

**Fig. 5:**
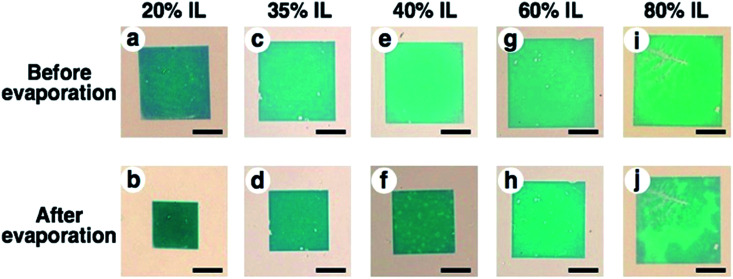
Optical microscopic images of nanodots arrays on IL gels with various degrees of swelling. Upper images are samples prepared through the solvent exchange to (a) 20%, (c) 35%, (e) 40%, (g) 60%, and (i) 80% IL solutions, and lower images are samples prepared with (b) 20%, (d) 35%, (f) 40%, (g) 60%, and (j) 80% IL solutions after evaporation. Scale bars represent 50 μm.

To obtain high resolution images by SEM observation as another approach, we prepared an ionic liquid gel. As mentioned above, electron microscopy is basically performed under high vacuum conditions and is not suitable for hydrogels due to the evaporation of the swelling solvent, causing shrinkage. Thus, we applied an ionic liquid (IL) as the solvent to swell the gel. An ionic liquid is a salt in the liquid state and has advantageous characteristics for SEM observation, such as non-volatility and high conductivity.^54,55^ From the view point of compatibility with the gel, 1-methylimidazolium hydrogen sulfate, which is a hydrophilic IL, was used in this study. To exchange the PAA gel solvent, hydrogels were immersed in IL solutions. While the size of PAA gels swollen with an IL (referred to as IL gels) did not actively change, the non-volatility of ILs provided stability to the gels without excess external solvent even under high vacuum conditions. This stability enabled the control of the degree of swelling without stimuli-responsiveness. In the experiment, we prepared mixtures of IL and water at various ratios (20, 35, 40, 60, and 80% of IL contents) for the IL gels. The exchange of the solvent from water to these IL mixtures was successfully performed and provided different sized gels swollen with the IL-water mixture (Fig. 5 upper images, shown as before evaporation). After removal of excess external solution, these gels were evaporated under vacuum to obtain IL gels containing no water. The prepared IL gels were referred to as *X*% IL gels (*X* represents the IL content of the original IL mixture) and their photos are shown in Fig. 5 lower images (b, d, f, h and j) as after evaporation. These photos are similar to those of the hydrogels in Fig. 3A. The removal of water from the IL gels was confirmed by the volume change of the gels before and after evaporation (Fig. S4 and Table S1†).

To confirm that the gold nanodot pattern on the IL gels was the same as that on the hydrogels, we performed AFM imaging and measured their extinction spectra. AFM images of IL gels with different gel sizes (*L* = 1.0, 1.1, and 1.3) were quite similar to those of the hydrogels (Fig. S5†). Further, their extinction spectra were comparable with those of the hydrogels, even though a few nm of peak shift were observed, probably due to differences in the dielectric constants of the surrounding media (Fig. S6†). These data support the notion that the gold nanodot patterns on the IL gels were the same as those on the hydrogels.

**Fig. 6:**
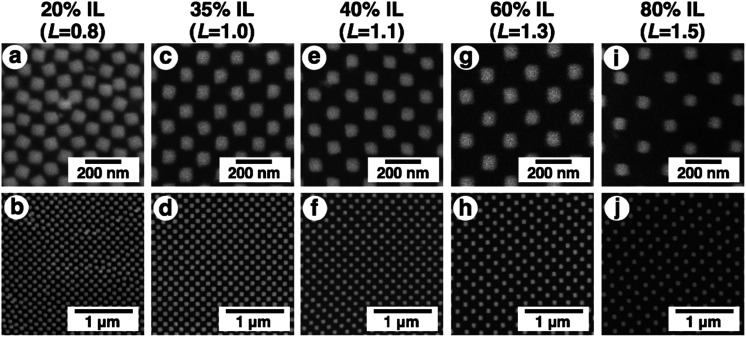
SEM images of IL gels with various degrees of swelling, 20% IL gel (*L* = 0.8) (a and b), 35% IL gel (*L* = 1.0) (c and d), 40% IL (*L* = 1.1) (e and f), 60% IL (*L* = 1.3) (g and h), and 80% IL (*L* = 1.5) (i and j). Upper images (a, c, e, g and i) are high magnification and lower images (b, d, f, h and j) are low magnification. One pixel corresponds to 2.7 (a), 3.8 (c), 3.1 (e), 4.7 (g), and 3.8 nm (i), respectively.

Then, the detailed structures were observed by SEM (Fig. 6). Due to the advantageous characteristics of the ILs, SEM observation was successfully performed without any compaction of the gel under high vacuum conditions or charge-up from electron beam exposure under no gold or carbon deposited state. These SEM images were quite similar to the AFM images except in terms of resolution (Fig. 4, 6, and S5†). The resolution was sufficiently high compared to the conventional observation on a silicon substrate (Fig. 2A and 6). A quick look at these images shows that their pattern changed uniformly except for *L* = 0.8. At *L* = 0.8, some rotation of the gold nanodots was observed, suggesting some lateral pressure similar to that on the hydrogel (Fig. 4a). For quantitative evaluation, the dot size as a diagonal length, the center-to-center distance between nanodots, and the gap distance between the nearest edges were measured based on these SEM images by ImageJ (Table 1). On these images, 1 pixel corresponds to 2.7–4.7 nm depending on the picture (details are shown in the legend for Fig. 6). When the gel size was the same as that just prepared (*L* = 1.0), which means the same size as the original structure on the silicon substrate, the center-to-center distance was quite similar, but the dot size and gap distance show *ca.* 6 nm differences compared to the original structure; that is, 6 nm shorter for the dot size and 6 nm longer for the gap distance. The dot sizes on the other gels (*L* = 0.8, 1.1, and 1.5, except 1.3) were also 6 nm shorter than that on silicon. These results indicate a small change in the shape of the gold dots occurring on their transfer to the gel or during exchange of the solvent to the IL. On the other hand, the small standard deviations of the dot sizes and center-to-center distances on the IL gels of various sizes, including *L* = 0.8, support the notion that homogenous structures remained despite the volume change in the gels. Even though there was a small shape change in or some rotation of the nanodots at *L* = 0.8, the uniformity of the dot pattern remained high on a several nm scale, which is close to the resolution limit on these SEM images, during the size changes in the gel (Table 1).

**Fig. 7:**
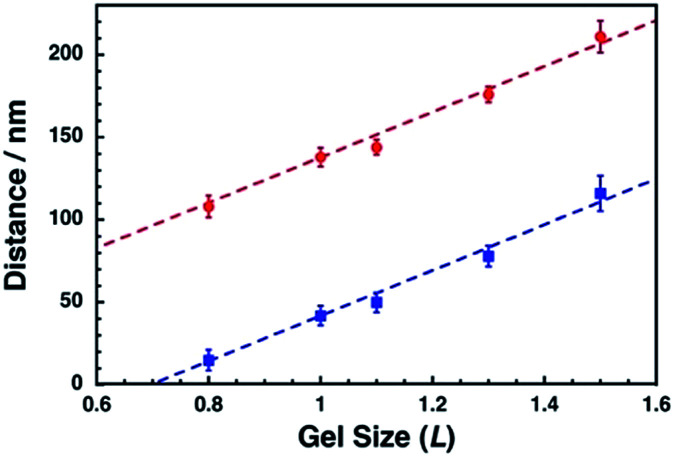
Comparison of center-to-center distances (red) and gap distances (blue) between those of our experimental results and theoretical calculations. The experimental results were shown as plots and theoretical calculations were shown as broken lines. Error bars represent the standard deviation.

To confirm the correlation between the dot pattern change and gel size change, we calculated the ideal distances for the center-to-center distance and their gaps and compared them with our results (Fig. 7). The shape of a nanodot was determined from the SEM image of *L* = 1.0. Then, the dots were uniformly placed with a center-to-center distance of 138 nm and a gap of 42 nm and their pattern was expanded or compacted uniformly (Fig. S7†). Fig. 7 shows that the observed center-to-center and gap distances were well fitted to the calculated theoretical curves. These results support the notion that a pattern of sub-100 nm dots can be actively tuned by the gel size change with nanoscale uniformity.

### Spectral analyses of actively tunable plasmonic nanoarrays

**Fig. 8:**
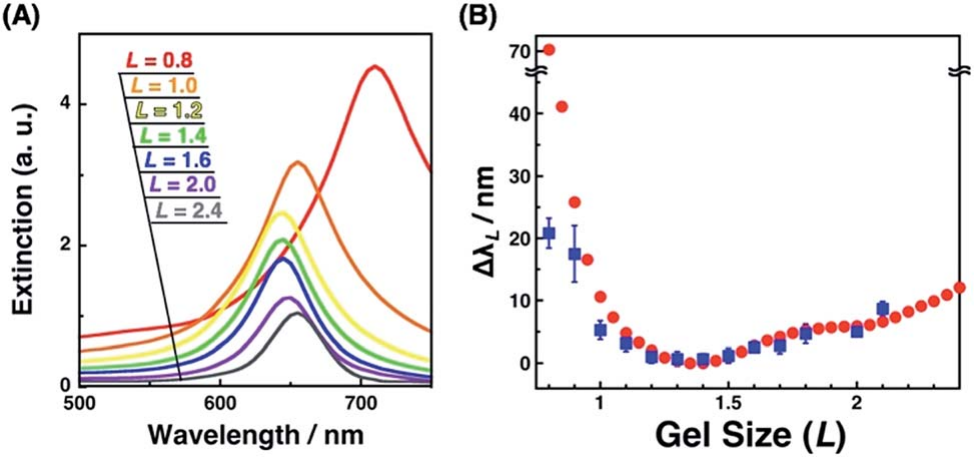
Extinction cross-sectional spectra of gold nanodot arrays with various distances calculated by FDTD simulation (A) and the plots of spectral shifts (Δ*λ*_*L*_) against the gel size (*L*) (B); Δ*λ*_*L*_ calculated from simulation (red circles) and Δ*λ*_*L*_ determined from experimental spectra of Fig. 3C (blue square). Error bars in (B) (blue) represent the standard deviation (*n* = 3–6).

Plasmonic spectra can be computationally simulated when the conditions, such as material, structure, and external environment, are defined. Here, we have successfully determined the nanostructures by SEM observation. As the surface plasmon is particularly sensitive to the shape or structure on a nanoscale and a small structural variance can cause critical plasmonic changes, a comparison of extinction spectra with simulation data is useful. Thus, the FDTD simulation was carried out. Simulated extinction spectra and Δ*λ*_*L*_ were quite similar to our experimental data (Fig. 3C and 8). Below *L* = 1.3, Δ*λ* exponentially grew in response to gel shrinkage, indicating the presence of well-known plasmon coupling effects. There are many reports of simulations that provide exponential spectral shifts depending on the gap distances between spherical nanostructures.^56,57^ On the other hand, the plasmon peaks interestingly showed a slight blue shift from *L* = 2.5 to 1.4. This blue shift is due to radiative dipolar coupling between the nanodots and retardation effects.^58^ As a result, gold nanodot patterns with moderate gaps showed a weaker surface plasmon compared to independently existing nanodots with sufficiently wide distances. Here, a good consensus between the experimental spectra of the gold nanoarrays on the hydrogel and simulations based on the SEM images of those on the IL gels supports the fact that our nanoscale images on the IL gels correspond to their structures on the hydrogels, and also that the small structural deformation of nanodots did not occur during the solvent exchange but during the transfer process.

Interestingly, only for *L* = 0.8 as the most compacted structure in this study, Δ*λ* showed a significant difference between the experimental and simulation data. That is, the experimental shift was too small compared to that of the simulation. As already mentioned above, there are some wrinkles on the gels observed by AFM for *L* = 0.8 (Fig. 4a) and some rotations of the gold nanodots were also observed by SEM (Fig. 6a), indicating lateral stress and strain. This rotation could explain some dispersions of the gap distances at *L* = 0.8 and also a smaller red shift compared to that assumed in the simulation data as the ideal structure change, even though the dispersion in the gap distance was quite small (Table 1). Further, these findings also suggest that even though there is a sufficiently strong lateral pressure to make wrinkles, the gold nanodots remain attached on the hydrogel maintaining a uniform pattern due to the strong interaction and systematic release of the stress by their rotation. Thus, this rotation is thought to be good news in that it supports the robustness of this large and repeatable active gap control system.

**Fig. 9:**
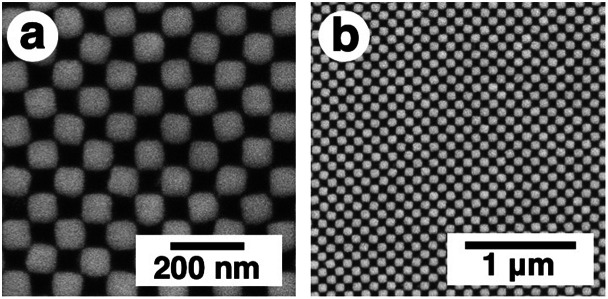
SEM images of 15% IL gel (*L* = 0.8) which was prepared with UV-oxone cleaning (a and b). Center-to-center distance and gap distance were 104 ± 3 nm (*n* = 100) and 7 ± 4 nm (*n* = 140), respectively.

One question that remains here is why there was a significant stress even though the gap distances remained over 10 nm for *L* = 0.8, causing smaller spectral shifts. Our careful SEM observation suggested the possibility that some EB resist remained at the edge of gold nanodots due to the insufficient removal. Thus, we performed UV–ozone cleaning after the lift-off process. As a result, we obtained large spectral shifts in shrunken states (*ca.* 60 nm at *L* = 0.8; Fig. S9A†). These large spectral shifts were well matched with the simulation results (Fig. S9B†). Further, the SEM results showed no distortion of the nanostructures even in the narrow gaps (Fig. 9). This result supports the good controllability of gap distances over a wide range on a nanoscale.

## Conclusions

In this study, we fabricated gold nanopatterns with sub-100 nm dots on the surface of poly(acrylic acid) hydrogels. Then, active tuning of the nanodot patterns on the hydrogels was confirmed by spectroscopic analysis and AFM observation. Although the AFM images provided clear phase images due to the differences in their characteristics and some information on array pattern changes, their resolution was not sufficient for the simulation of plasmonic spectra. Thus, we changed the solvent from water to an ionic liquid and performed SEM observation. Solvent-exchanged IL gels with various degrees of swelling were prepared through the exchange of a mixture of IL and water followed by evaporation of the contained water. Extinction spectra and AFM images supported the absence of any change in the nanopatterns on solvent exchange. SEM images proved the existence of very uniform pattern changes with changes in the size of the gels on a nanoscale. FDTD simulations calculated based on the SEM images were well matched with the actively tuned plasmonic patterns observed in water. As a result, our nanoscale imaging of the actively tunable plasmonic arrays on the hydrogels supports the notion that homogenous tuning of nanostructures can be performed by the volume change of hydrogels on a scale of several nm, regardless of the inhomogeneity of the polymer networks in the gels. Even if there is still some room for improvement in the resolution of this imaging, it may have no significance as there are some fluctuations including Brownian motion on the polymers on a several nm scale.^59^ Our approaches and results in this study strongly support the tremendous potential of the active tuning of nanostructures by hydrogels and open future applications to biosensing using surface enhanced Raman scattering.

## Conflicts of interest

There are no conflicts to declare.

## Supplementary Material

NA-001-C8NA00404H-s001
