# Supplementary material for: Nanoscale uniformity in the active tuning of a plasmonic array by polymer gel volume change
Source: Nanoscale Adv. 2019 Mar 4;1(5):1731–9. doi: 10.1039/c8na00404h (PMC9418027; doi:10.1039/c8na00404h)
Supplement: NA-001-C8NA00404H-s001 [file NA-001-C8NA00404H-s001.pdf]

Electronic Supplementary Material (ESI) for Nanoscale Advances.
This journal is © The Royal Society of Chemistry 2019

Nanoscale uniformity in the active tuning of a plasmonic array using polymer gel volume change

Satoru Hamajima,[†a] *Hideyuki Mitomo,*[*†b,c] *Takeharu Tani,*[d] *Yasutaka Matsuo,*[b] *Kenichi Niikura,*[e] *Masayuki Naya*[d] and *Kuniharu Ijiro*[*b,c]

a Graduate School of Chemical Sciences and Engineering, Hokkaido University, Kita 13, Nishi 8, Kita-Ku, Sapporo 060-8628, Japan

b Research Institute for Electronic Science, Hokkaido University, Kita 21, Nishi 10, Kita-Ku, Sapporo 001-0021, Japan

c Global Station for Soft Matter, Global Institution for Collaborative Research and Education, Hokkaido University, Kita 21, Nishi 11, Kita-Ku, Sapporo 001-0021, Japan

d FUJIFILM Corporation, Ushijima, Kaisei-Machi, Ashigarakami-gun, Kanagawa 258-8577, Japan

e Department of Applied Chemistry, and Graduate School of Environmental Symbiotic System Major, Nippon Institute of Technology, Miyashiro, Saitama 345-8501, Japan

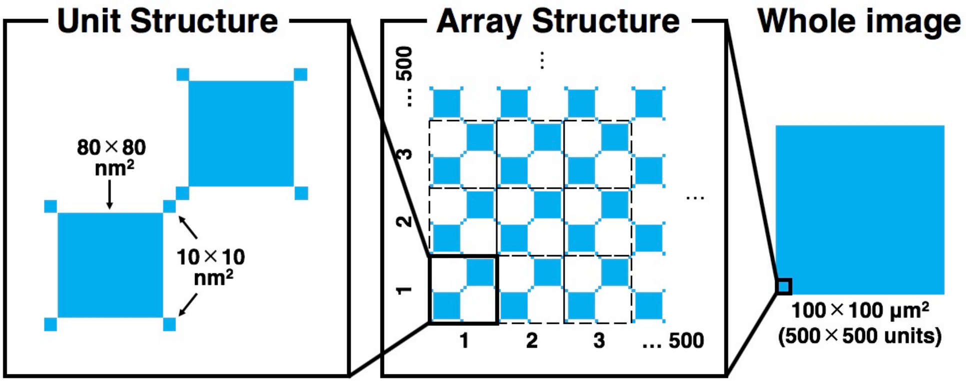

Figure S1. Original CAD design for EB lithography. Blue areas represent EB exposed positions for gold deposition.

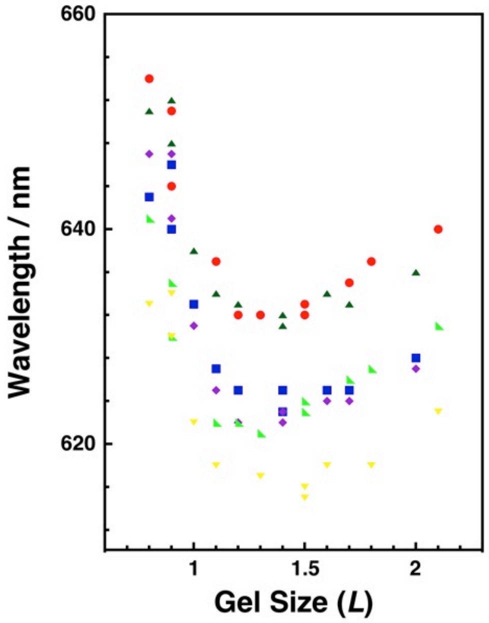

Figure S2. Plots of the spectral peak of each sample against gel size (*L*). Different symbols were used for each sample (n=6).

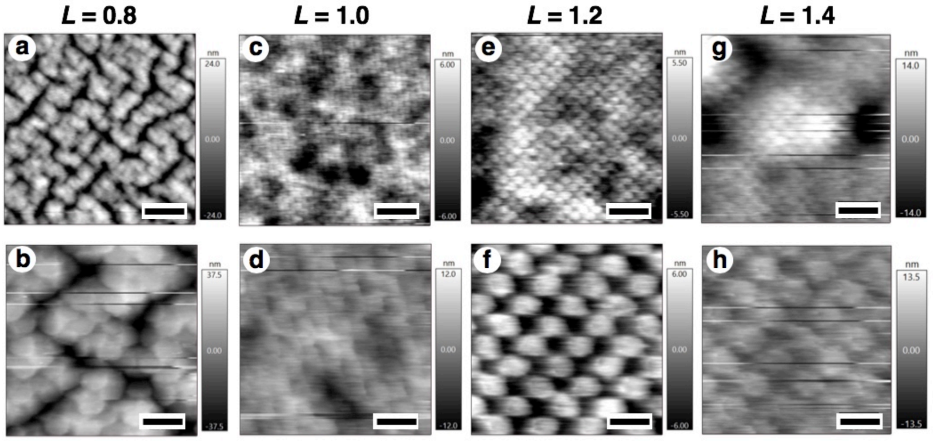

Figure S3. AFM height images of the surface patterns on hydrogels of various sizes, L=0.8 (a, b), L=1.0 (c, d), L=1.2 (e, f), and L=1.4 (g, h). Scale bars represent 600 nm in the upper images (a, c, e, g) and 200 nm in the lower images (b, d, f, h).

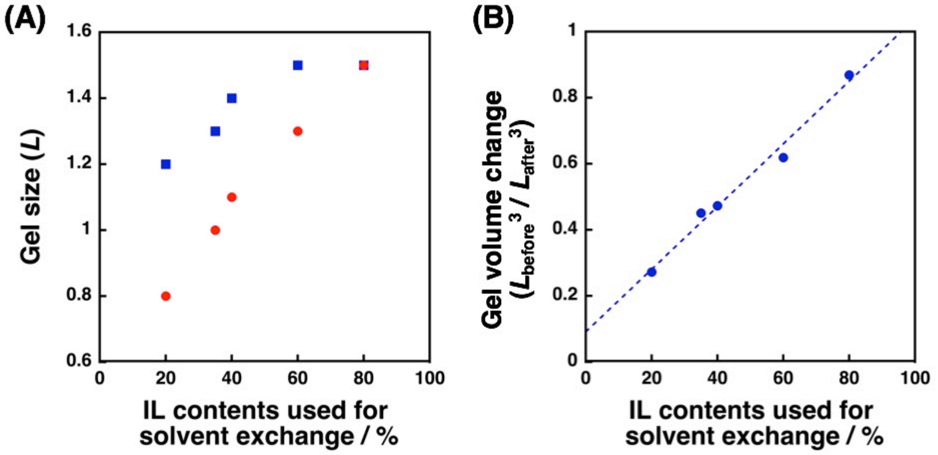

Figure S4. Gel size (A) and gel volume change rate (B) against IL content used for the solvent exchange. (A) Blue squares correspond to before evaporation and red circles correspond to after evaporation.

Table S1. The weight of added water, added ion liquid, residue after evaporation, and residual water as the difference between residue and added IL

Added Water (g)	Added IL (g)	Residue after evaporation (g)	Residual Water (g)
4.013	1.490	1.489	-0.001
4.024	1.499	1.502	0.003
3.977	1.464	1.468	0.004

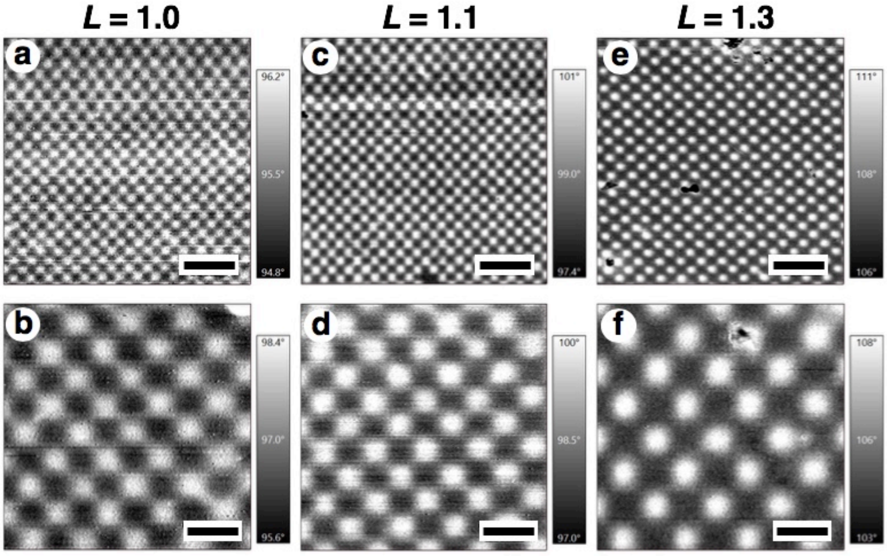

Figure S5. AFM phase images of the surface patterns on IL gels of various sizes, *L*=1.0 (a, b), *L*=1.1 (c, d), and *L*=1.3 (e, f),. Scale bars represent 600 nm in the upper images (a, c, e) and 200 nm in the lower images (b, d, f).

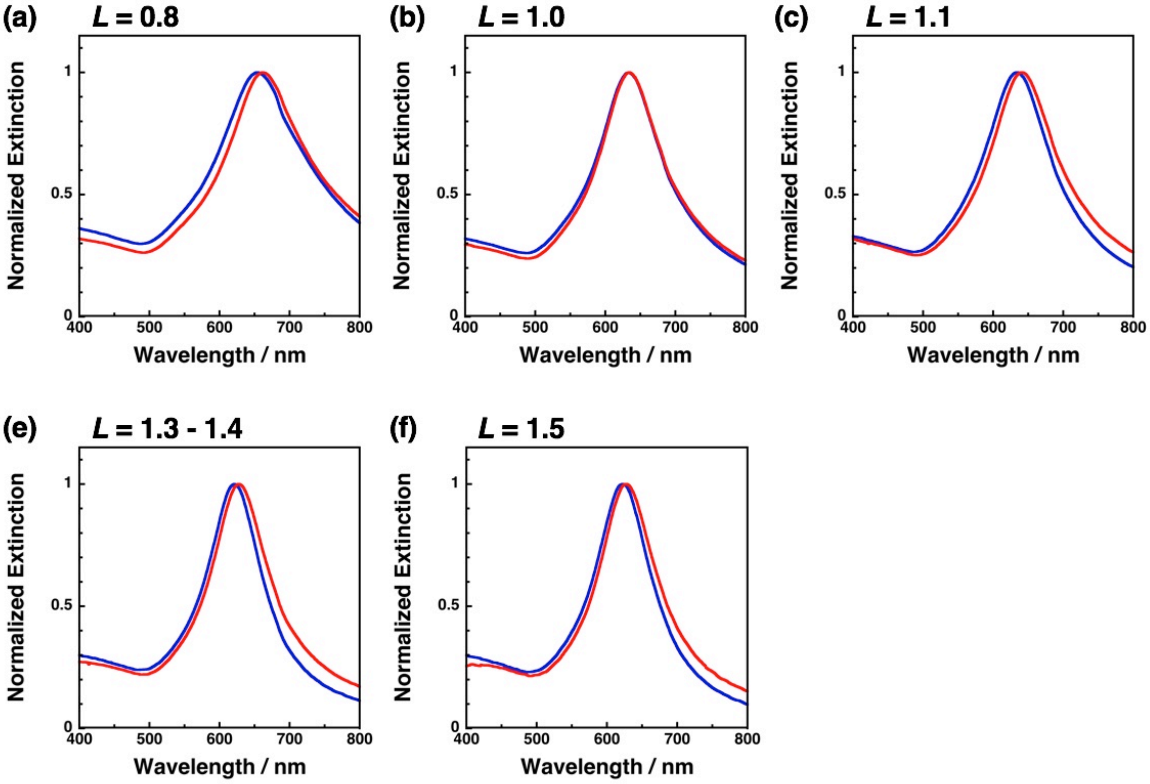

Figure S6. Normalized extinction spectra of hydrogels (blue) and IL gels (red) of various gel sizes (L). (a) L=0.8, (b) L=1.0, (c) L=1.1, (d) L=1.4 for hydrogel, L=1.3 for IL gels, and (e) L=1.5

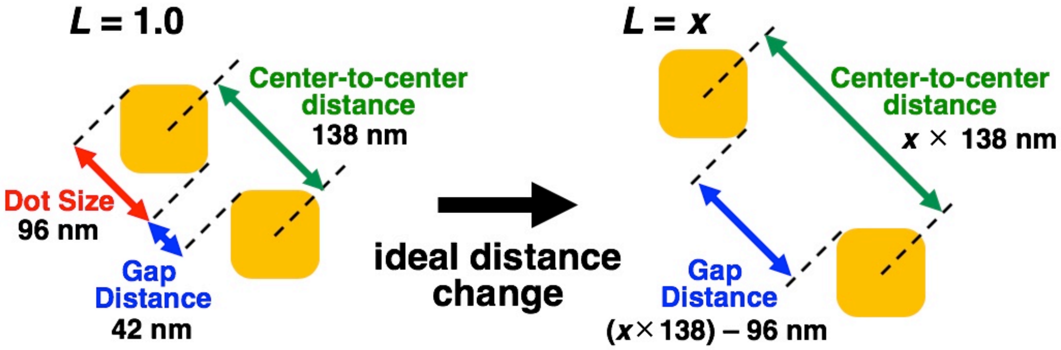

Figure S7. Schematic illustration of the ideal distance changes in gold nanodots with gel size (L) change.

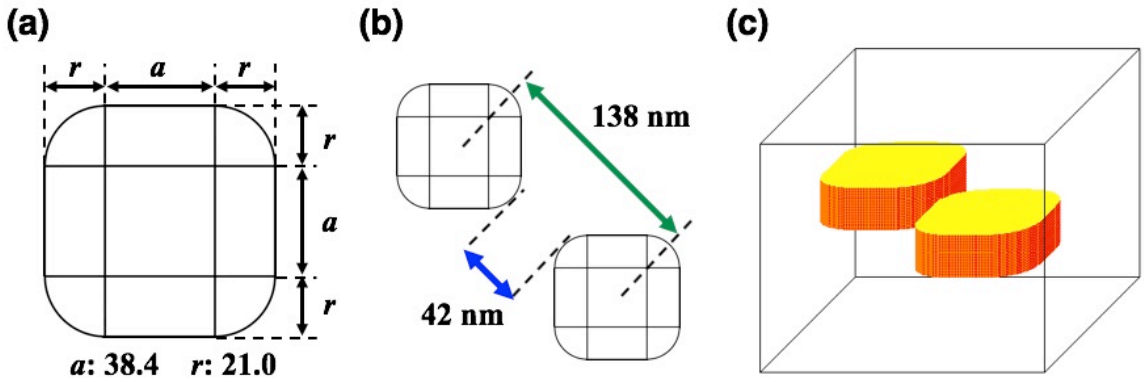

Figure S8. Schematic images of a defined nanodot (a) and their distances (b), and 3D images of gold nanodots as a unit structure (c) for the simulations.

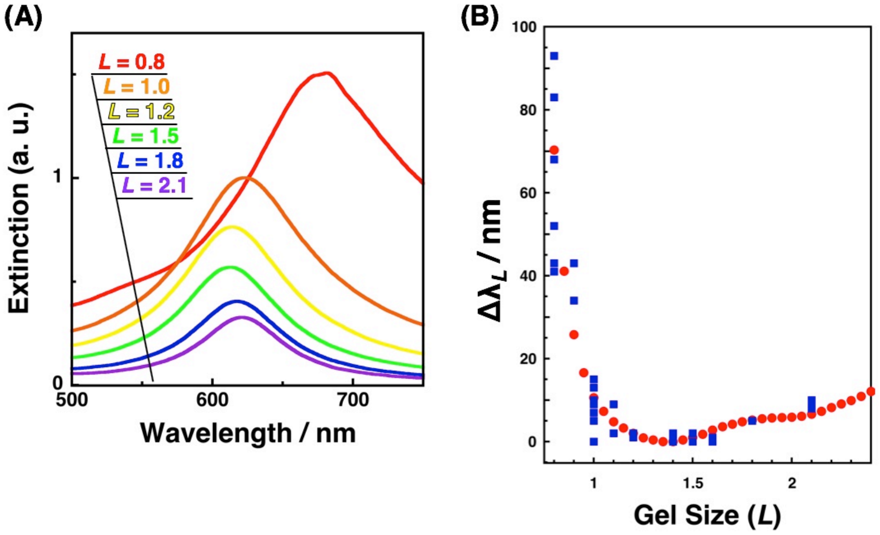

Figure S9. (A) Extinction spectra of nanodot arrays, which were prepared with UV-ozone cleaning, on hydrogels with various degrees of swelling and (B) the plots of spectral shifts ($\Delta\lambda_L$) against gel size (L); $\Delta\lambda_L$ calculated from simulation (red circles) and $\Delta\lambda_L$ determined from experimental spectra of the samples prepared with UV-ozone cleaning (blue square).
